# Climate finance and women-hunger alleviation in the global south: Is the Sub-Saharan Africa case any different?

**DOI:** 10.1371/journal.pone.0290274

**Published:** 2024-02-05

**Authors:** Isaac Doku, Andrew Phiri

**Affiliations:** 1 Department of Economics Education, University of Education, Winneba, Kumasi, Ghana; 2 Department of Economic Science, Nelson Mandela University, Gqeberha, South Africa; Newcastle University Business School, UNITED KINGDOM

## Abstract

To unearth the influence of climate finance (CF) on women-hunger alleviation in Sub-Saharan Africa (SSA), the study used unbalanced panel data for 43 SSA countries for the period 2006–2018. Data was analysed using system-GMM to deal with the endogeneity problem inherent in the model, among other panel regression estimators. Also, the sensitivity of the estimates was carried out using panel fixed effect quantile regression. The findings showed that CF and its components have a significant effect on women-hunger alleviation in SSA, apart from FDI. Further, control of corruption also showed a significant women-hunger alleviation impact. For the climate variables, areas in SSA with higher temperature are more likely to experience worsened women-hunger. Based on the findings, the study recommends that SSA countries need to strengthen their fight against corruption. More so, donors should extend CF as financial aid or support to government budget, due to their potential of alleviating women-hunger.

## 1 Introduction

Women empowerment in the agricultural value chain, access to resources and trade has the potential of speeding up the realisation of Sustainable Development Goal-2 (SDG-2)-end hunger-and the Malabo commitments. The Malabo declaration imbibes all African countries to end hunger by 2025. This agenda is realisable only if the problem of gender inequality in food production is ameliorated [1]. The gender equality dimensions of the Malabo declaration, focuses on women owning 30 percent of documented land and accessing 50 percent of finance to end hunger. In that same vein, SDG-2 postulates that all forms of hunger should be ended by 2030, including addressing the nutritional needs of pregnant and lactating women and adolescent girls worldwide (Goal 2 | Department of Economic and Social Affairs (un.org)). It is estimated that 2 billion people worldwide—representing one in every four persons—have no access to enough reliable nutritious and safe food. In 2020, 690 million people were estimated to be chronically hungry, a figure expected to increase by 130 million due to COVID-19 [1, 2].

[3] opined that women and girls account for 60% of all those facing chronic hunger worldwide. Meanwhile, agriculture is the mainstay of over 1.4 billion women worldwide living in rural areas. In the global South, approximately 43% of the labour force are women, with 66% of them being livestock keepers [2, 4]. Yet, women have very little access to and control over land and other productive resources, market and education as compared to men. For instance, women form only 13% of agriculture land-owners globally [5]. The gender situation seems more interesting in Africa, where women form on average 60% of the agricultural labour force and play a very significant role in hunger alleviation by growing up to 89% of what the family consumes [6, 7]. A problem that is gradually creeping into the agricultural sector in the global south is that men are drawn away from farming and increasing the role of women in growing food for family income diversification. According to [8], this is known as the ‘feminisation of agriculture’. Linked to the ‘feminisation of agriculture’ is the problem of ‘feminization of poverty’. ‘Feminisation of poverty’ reinforces the idea that the majority of the world’s poor and hungry ones are females [4, 7].

Interestingly, for all women in the reproduction age, one-third of them suffer from anaemia that is attributable mainly to food deficiency (Goal 2 | Department of Economic and Social Affairs (un.org)). The Agriculture Development Bank in 2013 pointed out that 55% of global gains achieved in reducing hunger is directly attributable to progress made in women’s education and levels of equality [9]. It will cost the world $23,620 per person and global-equivalent to an aggregate of $160.2 trillion if the world fails to address the cost of gender inequality [10].

The number of hungry ones continues to show an upward trend worldwide. For instance, the number of food insecure people in Latin America has tripled; doubled for Western and Central Africa; and a 90% increase in Southern Africa [1]. The United Nations posits that a quarter of the world’s hungry ones—denoting 220 million people—reside in sub-Saharan Africa (SSA), where a looming danger of humanitarian food crisis is imminent [4]. It is interesting to note that SSA women represent more than a fifth of the global South’s agricultural workforce-denoting 500 million women and are often subsistence farmers that grow crops for consumption and not to sell [4]. These statistics show that women’s labour productivity is essential in the food sector to end hunger, especially at a time when climate change is adversely impacting crop production. However, the economic contribution of women in ending hunger has been overlooked in SSA. For that matter, it is vital to deal with hunger, food security, and rural poverty in a holistic manner. This must be done by integrating the mitigation and adaptation of climate change, biodiversity, and gender equality. This is an approach relevant to rural women empowerment in SSA.

The exacerbating effect of climate change on hunger cannot be overemphasized in Africa- a region where only four percent of farms are irrigated [11, 12]. For instance, rain-dependent crops like peanuts and millet are experiencing reduced productivity attributable to sporadic rainfall. In other to circumvent the problem of crop failure, women in Africa are beginning to grow drought-resistant rice among other cereals. If SSA governments and the international community commit to helping women grow climate-resilient food such as millet and peanut, it will help deal greatly with hunger among the vulnerable group (i.e. women and children). Millet, for instance, is rich in protein and calcium, important nutrients relevant for growing children and pregnant or lactating mothers. Peanuts also contain very high protein relevant for muscle and brain development [13]. This indicates that, with a clear focus by SSA on mitigating and adapting to climate change in the agriculture sector, women-hunger will be reduced to the barest minimum.

Climate change is worsening hunger among women, men, and children in SSA; through the vagaries of the weather, extreme temperature, sporadic rainfall, and an upsurge in the intensity and frequency of climate-induced disasters such as droughts, floods, cyclones and storms. It is estimated that globally, climate-induced disasters cost billions of dollars as a result of economic damage and the destruction of lives and livelihoods of billions [14–17]. If no climate action is taken, the amount needed to address climate change impact in the future will grow exponentially. As of now, the projected cost globally is $69 trillion by 2100 if the 2°C threshold is crossed [18]. However, the Intergovernmental Panel on Climate Change (IPCC) [18] mentioned that the tipping point of 1°c has already been crossed since 2017. Detailed research however projects that women are expected to be highly affected by climate change than men because they are the most vulnerable and marginalised group. Especially, women in SSA need to contend with limited access to finance, weak land tenure system and persistent social inequality hampering their agricultural capacity and productivity.

As a result of the foregoing, Article 4 of the 1992 UNFCCC posits that developed countries should provide ‘new and additional’ financial flows (climate finance) to help developing countries mitigate and adapt to climate change- termed climate finance [16, 19–25]. Climate finance has been mentioned by the IPCC and UNFCCC, as a very important tool in addressing the gender inequality problem. If the climate finance architecture is designed to deal with gender issues; it will promote inclusive, equitable, and just climate actions to alleviate hunger and achieve food security. The good news is that climate finance donors have increased their gender targets in climate actions. This is evidenced in the increase in climate finance targeting gender by 55% in 2014 from 2010 [16]. Yet in 2014, gender accounted for only 31% of total climate finance flows-representing $8 billion-from major donors [16].

In the hunger literature, several studies have looked at climate change and hunger or food security among women in SSA [1, 7, 13, 26–30]. A few studies tried looking at climate finance and hunger [31, 32], and others tried explaining the need to channel enough climate finance to deal with gender equality [9]. To the best of our knowledge, no study has empirically tested the influence of climate finance on women-hunger in SSA. This study contributes to the hunger literature in two folds. Firstly, by determining the influence of climate finance in alleviating hunger among the most vulnerable gender in SSA, by dealing with the problem of ‘feminisation of poverty’ which is closely linked to hunger in the sub-region. Secondly, to find out whether adaptation or mitigation finance better help in women-hunger alleviation in the region most susceptible to the exacerbating effect of climate change.

## 2 Literature review

In gender literature, feminist theorists are the major theories that try to understand the nature of gender inequalities in society [33]. They believe that before the 1970s most scientific studies focused on male-only samples, and the results generalized to cover women and children [34]. However, the effect of the environment, accessibility of finance and land acquisition on poverty and hunger differ between men and women. Especially in most developing countries where women make sure to serve their husbands and children before they attend to themselves. More so, the environment worsens women poverty and hunger through health-related problems. Women are exposed to environmental-related health hazards such as cancer, asthma, lead poisoning, reproductive disorders, and other types of cancers [34]. Also, the livelihood of most women is dependent on climate-sensitive sectors such as subsistence agriculture, forestry and water [13]. In addition, women have less capacity and resources compared to men in mitigating and adapting to climate change.

Some prior studies indicate that women are 14 times more likely to perish in climate-related disasters than men [35, 36]. An instance was the 1991 cyclone and flood in Bangladesh, where 90% of the victims were females. Some reasons given included the fact that early warning signals were not sent to women who were predominantly caregivers at home; most of them lacked swimming skills; some of the women tried escaping the floods holding infants and towing elderly family members, whereas husbands escaped alone [34]. This is a common situation in the global south like SSA too, although not the case in the global north or developed countries. This affirms the existence of the cultural feminist theory in the global south. The cultural feminist theory asserts that women and men experience the social world differently. This is largely due to the differences in values associated with womanhood and femininity in culture [33]. Explaining the reason why women hunger is a major problem in the global south, especially SSA.

The existence of feminism in agriculture, poverty, and hunger is further affirmed by studies such as [37]. They found that women farmers have lower rates of agricultural productivity than men farmers. This study was carried out among five countries in SSA-Ethiopia, Malawi, Rwanda, Uganda, and United Republic of Tanzania. The study sort to show that gender gaps exist even in agricultural productivity. These gaps arise not because women are less efficient farmers but because they experience inequitable access to agricultural inputs; including family labour, high-yielding crops, pesticides and fertilizers. To close the gender gap, efforts must be made to equalise women’s access to agricultural inputs such as time-saving equipment [37, 38]. These affirm the existence of the structural oppression theory of feminism by Freidrich Engels and Karl Marx [33]. The proponents argue that the working class and powerless (like women) are exploited due to capitalism. The capitalist which mostly involves men oppresses the women due to their lack of power. To an extent, funds geared toward poverty alleviation and hunger extended to women are taken by their husbands, leaving them poor. Women do so to ensure peace and stability in the family. Based on that, it is expected that climate funds extended to women will not necessarily help alleviate poverty but in most cases funds are usurped by their husbands.

It is established in the hunger literature that, if women (constituting 43% of the agricultural labour force in developing countries) have equal access to finance as men, food production could rise by up to 4%, potentially reducing the number of undernourished people by 12–17% [7, 39, 40]. Sadly, in some African rural communities, women are not allowed to have their own bank accounts, negotiate with suppliers or use other financial services. Other gender-based constraints include comparatively diminished access to technology, services, modern inputs, and markets. Across Africa, women also tend to have smaller plots and less control over labour and land. Some prior studies argue that, until the world pays greater attention to gender relationship in fund allocation and loan reimbursement, even funds targeting women may land in the hands of men [16]. For instance, Goetz and Sen Gupta [41] found that men use female household members in securing loans, due to women’s higher loan repayment rates. When women fail to obtain loans desired by male relatives, it breeds tension at home [42].

The single most important determinant of food security in the hunger literature is gender equality, which is capable of contributing significantly to a country’s growth [43, 44]. An example is a study by Smith and Haddad [45]. The study found that 43% of the hunger reduction which occurred between 1970–1995 among developing countries, is attributable to the progress made in women’s education. A value equivalent to the combined effect on hunger reduction of increased food availability (26%) and improvements in the health environment (19%) for that same period [45]. In that same study, an additional 12% of hunger reduction was attributable to an increase in the life expectancy of women. In short, a combined total of 55% of hunger reduction among developing countries during the period, were attributable to the improvement of women’s situation within society [45]. The global hunger index was used to compare hunger among countries globally. The findings revealed a significant correlation between hunger and gender inequalities [46]. Implying that, countries with very high hunger index are those with severe gender inequalities [43, 47–53, 55].

A tool that can be effective in dealing with gender inequalities in the global south is Climate finance (CF). The reason is that designing CF to promptly respond to gender-related issues tends to enhance equitable and inclusive climate action to enhance sustainable development. As a result, OECD countries and other CF donors are increasingly targeting the gender component of any climate action by a country [54]. The OECD [54] report intimates that total global aid targeting gender and climate change sour upward by 55% in 2014 from 2010. The report further indicated that gender accounted for 31% -equivalent to USD 8 billion- of CF provided by major donors that are members of the Development Assistance Committee (DAC) [14, 15, 17]. This study contributes to both hunger and feminism literature, by finding out whether CF so far received by SSA countries is helping realise women-hunger alleviation. For that matter, climate finance is expected to reduce women-hunger as their haemoglobin levels rise. The null hypothesis to be tested in this study is:

*H_o_: Climate finance reduces women-hunger in SSA*

## 3 Methodology

### 3.1 Data

To better estimate the impact of climate finance on women-hunger alleviation in SSA, data for all SSA countries must be used. However, some SSA countries have very large missing data points for the study period; these include Cape Verde, Equatorial Guinea, Sao-Tome and Principe, Seychelles, and Somalia. This leaves us with an unbalanced panel data of 43 countries in SSA for the period 2006–2018 used for the estimation. The estimation was carried out using System Generalised Method of Moment (SYS-GMM), Pooled Ordinary Least Squares (POLS) and panel fixed effect (FE) models. A robustness check was carried out using panel fixed effect quantile regression.

#### Dependent variable

The main dependent variable of the study is women-hunger (a variable which is very cumbersome to measure in practice, since hunger is felt at the individual level). For that matter, we employed a variable used by FAOSTAT in determining food utilisation by women under food security- the “prevalence of anaemia among women of reproductive age” (age ranges from 15 to 49 years). Although the variable was collated from FAOSTAT, its main source is from the World Health Organisation (WHO) Global Health Observatory data repository. The variable was computed by looking at the percentage of women in the age bracket of 15–49 years, which have haemoglobin levels less than 120 g/L for non-pregnant and lactating women and less than 110 g/L for pregnant women. Low haemoglobin and iron deficiency is a serious consequence of hunger among women of reproductive age and children. Women-hunger is highly prevalent among three of the regional blocs: Economic Community of West Africa State (ECOWAS), Economic Community of Central Africa State (CEMAC) and Southern Africa Development Community (SADC), with all having values above the overall mean of 41 percent (refer to Table 2). Only the East Africa Community (EAC) has a women-hunger score below the mean (refer to Table 1).

**Table 1:** Descriptive statistics for regional blocs in SSA.

Variable	ECOWAS	EAC	CEMAC	SADC
**Women-hunger**	48.667	31.357	48.747	48.049
**Temperature**	28.702	23.522	25.053	19.992
**Climate finance**	138,418	323,179.9	84,349	133,595.2
**Adaptation**	84,115.5	190,928	52,226.28	54,582.41
**Mitigation**	54,302.5	132,251.9	32,122.72	79,012.83
**Aid**	88.151	69.323	42.637	68.527
**FDI**	8.17e+08	7.79e+08	6.27e+08	1.21e+09
**Gov’t-Spending**	6.178	7.364	2.584	4.995
**Population**	20.361	27.718	17.615	14.503
**GDP per capita**	2840.4	3334.7	8154.7	7565.512
**Corruption Control**	0.289	0.274	0.146	0.4002
**Food Production**	35.385	36.283	40.555	42.513
**Food import**	68.971	88	11.647	12.727
**Agricultural Land use**	48.891	59.622	23.483	53.745
**Irrigation**	3.092	12.706	0.727	4.086

#### Independent variables

The main independent variable of the study is climate finance (CF) and other financing variables such as aid, FDI, and government expenditure. In this study, CF is the ‘new and additional’ financial flows from developed countries to help developing countries mitigate and adapt to climate change in million USD. CF data was sourced from the Organisation of Economic Co-operation and Development (OECD), Donor Assistant Commitment (DAC) climate-related development finance. From Table 1, it is clear that EAC received the largest sum of climate finance (i.e. 323 million per year on average), followed by the ECOWAS region. Sadly, the CEMAC region is the least recipient of climate finance in SSA. Further, climate finance data is segregated into adaptation and mitigation. For mitigation, SADC region is the second largest recipient after EAC (refer to Table 1). Both mitigation and adaptation finance extended to SSA show very high variability across the region, as indicated by a very high standard deviation (146378.9 for adaptation finance and 141426.4 for mitigation finance) (refer to Table 2). The reason may be due to differences in the vulnerability levels of all countries in the sub-region. If climate finance is helping achieve women-hunger alleviation contrary to the dictates of the structural oppression theory of feminism, then we expect a significant negative effect on women-hunger. To ensure that climate finance extended to SSA is not siphoned or becomes fungible, we include a corruption control variable (COC) which is sourced from Notre-Dame Global Adaptation Index (ND-GAIN). From Table 2, it can be seen that corruption control in SSA is very low (Average of 0.27), although the fight is better in the SADC region (With an average of 0.4) there is more room for improvement for all countries in SSA.

**Table 2:** Descriptive statistics.

Variable	Mean	Std. Dev.	Min	Max
**Women-Hunger**	41.308	11.103	19	61.5
**Climate Finance**	199,384.98	284,664.7	59.839	2,428,679
**Adaptation**	101,400	146378.9	3.654	1226671
**Mitigation**	73,395.98	141426.4	1.347	1202008
**Aid**	70.736	70.211	0.463	663.711
**FDI**	8.47e+08	1.60e+09	-7.4e+09	1.00e+10
**Gov’t-Spending**	5.627	4.659	0.145	28.88279
**Population**	21.310	30.361	0.5	195.9
**GDP per capita**	4711.502	6117.886	761.5	41249.4
**Control-of-Corruption**	0.273	0.144	0	0.665
**Food Production**	37.563	24.079	4	215
**Food Import**	54.660	118.797	0	775
**Agricultural Land Use**	47.362	19.858	8.154	80.920
**Irrigation**	5.471	11.374	0.1	78.9

Other financing variables-such as aid, FDI and government expenditure are included in the study to cover for the broader definition of climate finance as stipulated by the Paris Agreement. The broader definition explains climate finance as “…financial resources provided to assist developing countries concerning both mitigation and adaptation.” (UNFCCC, 2015: pg. 13). In light of that, the Paris Agreement enjoins developed countries to support developing countries to mitigate and adapt to climate change, and at the same time called for, and encouraged the broader approach in understanding climate finance. This includes climate finance from the private sector via foreign direct investment (FDI); governments nationally determined contributions to climate change through government expenditure (Gov’t-Spending), and other forms of aid that helps mitigate and adapt to climate change. Data on FDI and aid are sourced from the World Development Indicators (WDI) and Government Spending from FAOSTAT. Our a-prior sign for all the climate finance variables is expected to be negative- to indicate a hunger reduction effect. Majority of FDI flows to the SADC region compared to anywhere else on the continent (averaging over 1.2 billion annually). CEMAC region continues to be the least recipient of FDI among the other climate finance variables (refer to Table 1). In this study, aid consists of per capita Overseas Development Assistance to each country in constant USD. From Table 2, an average of USD 70 per person is extended to SSA. ECOWAS has been the highest recipient of aid per capita with CEMAC region being the least recipient throughout the study period (refer to Table 1). Since the focus of this study is on women-hunger, Government spending looks at government’s annual expenditure on agriculture as a percentage of total expenditure. All governments in SSA spend averagely only 5 percent of their total expenditure in agricultural development (refer to Table 2). This value is far lower than that needed to achieve the Malabo declaration 2025. EAC is the area that spends the highest percentage of their total expenditure on agriculture (i.e. 7 percent) yet is still woefully inadequate.

Next, to establish the effect of climate change on women-hunger, temperature variable is included in the model. This variable is chosen instead of precipitation and rainfall due to the dominant climate of SSA. Secondly, including both rainfall and temperature in the model will create some noise. Temperature measures mean annual temperature for each country in centigrade, sourced from World Bank Climate Change Knowledge Portal (WBCCKP). ECOWAS is the most heated place in the sub-region.

To be sure of whether an increase in economic growth and agricultural productivity influences women-hunger in the face of climate change, we included GDP per capita, food production (FP), import of food (FI), agricultural land use (AGL) and irrigation. Apart from AGL which is sourced from WDI, the rest of the variables are collated from FAOSTAT. GDP per capita is a measure of welfare or poverty level and computed as GDP divided by the population of a country in constant 2010 USD. CEMAC region has proven to have the highest GDP per capita annual average of 8154-which is a value almost double the region’s average of 4,711. Food production in this study is the per capita food production variability variable from FAOSTAT, which is computed as the “food net per capita production value in constant 2004–2006 international USD”. The variable is included to find out to what extent does unstable food supply influences women-hunger in SSA. SADC region has very high food production variability, a problem that may stem from the poor rainfall pattern in the region. However, the ECOWAS region has seen a more stable FS which may be attributable to a more stable temperature in the area. Trade data in this study is the value of food imports in total merchandise exports, computed as a percentage of food imports over total merchandise export by FAOSTAT. From Table 1, it is indicative that EAC spends averagely 88 percent of its total merchandise export on importing food. This explains why much climate finance is extended to the area, due to high food insecurity which calls for high food import in the sub-region. Agricultural land use is computed as agricultural land expressed as a percentage of total land area, sourced from WDI. SSA has used an average of 47 percent of its land for agricultural purposes, with EAC using an average of 59 percent. Finally, irrigation is computed as the percentage of arable land equipped for irrigation and sourced from FAOSTAT. It is a measure that looks at the vulnerability of the agricultural sector to climatic shocks including water stress. Only 5 percent of SSA’s land is equipped for irrigation, yet EAC members used 13 percent of their arable land for irrigation, the highest in SSA. The least is by the CEMAC region which uses 0.7 percent of arable land for irrigation.

### 3.2 Model specification and estimation technique

In the hunger literature, hunger is modelled as a production function-either as a translog production function or a Cobb-Douglas production function. This is due to its correlation with food production [32, 55, 56]. Following the dynamic model proposed by [38], that modelled hunger of country (i) at a specific time (t) as an unobserved latent variable (y_it_); in this study, variable (y_it_) represents women-hunger in each country for a particular year.


$$Y_{it}=X_{it}B+{Ɛ}_{it}$$ (1)

The *X*_*it*_ vector constitutes macroeconomic variables like climate finance variables (climate finance, aid, FDI, Gov’t-Spending), climate-related variables (Rainfall and temperature) and agricultural productivity variables (Food production, food import, agricultural land use and irrigation), and Ɛ_it_ stands for the error term with zero mean, constant variance and normally distributed. Following Rodgers baseline model specified in Eq 1, two main equations were written; Eqs (2) and (3) to estimate the impact of climate finance on women-hunger in SSA. Eq (2) looks at climate finance on women-hunger, the CF variable is based on the narrow definition of climate finance. In model (3), the study employed the broad climate finance definition- by including FDI, aid and Gov’t spending in the model. This was to find out whether they will influence women-hunger in SSA.


$$\begin{array}{ll} Women-hunger_{it}= & \psi_{1}+\psi_{2}Women-hunger_{it-1}+\;\;\psi_{3}Temperature_{it}+\;\psi_{4}Population_{it}+\\ & \psi_{5}GPCY_{it}+\;\psi_{6}Control-of-Corruption_{it}+\;\psi_{7}Food\;Production_{it}+\\ & \psi_{8}Food\;Import_{it}+\psi_{9}Agricultural\;Land\;use_{it}+\;\psi_{10}Irrigation_{it}+\\ & \psi_{11}log\;of\;Climate\;Finance_{it}+{Ɛ}_{it} \end{array}$$ (2)

$$\begin{array}{ll} Women-hunger_{it}= & \psi_{1}+\psi_{2}Women-hunger_{it-1}+\;\psi_{2}Rainfall_{it}+\;\psi_{3}Temperature_{it}+\\ & \psi_{4}Aid_{it}+\;\;\psi_{5}FDI_{it}+\;\psi_{6}Gov^{\prime }t-Spending_{it}+\;\;\psi_{7}Population_{it}+\\ & \psi_{8}GPCY_{it}+\;\psi_{9}Control-of-Corruption_{it}+\;\psi_{10}Food\;Production_{it}+\\ & \psi_{11}Food\;Import_{it}+\;\;\psi_{12}Agricultural\;Land\;Use_{it}+\;\psi_{13}Irrigation_{it}+\\ & \psi_{14}\;log\;of\;Climate\;Finance_{it}+{Ɛ}_{it} \end{array}$$ (3)

Eqs 2 and 3 are estimated using both static and dynamic panel regression models to determine the influence of climate finance on women-hunger alleviation in SSA. For static panel data analysis, fixed effect (FE) and Pooled Ordinary Least Squares (POLS) panel data analysis. FE models are very important on the theoretical basis since they take care of the time-invariant heterogeneity across countries, and also provide robust results to omitted variable biasedness [57]. The relationship between climate finance and women-hunger is expected to be bidirectional. In the sense that much-gendered climate finance will be extended to countries with a higher risk of women-hunger, or countries with a greater number of hungry women are more likely to attract enough climate finance. This relationship is more likely to generate an endogeneity problem but can be dealt with by using dynamic panel regression such as system generalised method of moment (SYS-GMM). Further, dynamic panel regression model (Specifically SYS-GMM) was employed in this study due to large number of cross sections (43 countries), with a relatively shorter period (2006–2018). Finally, the inclusion of a lagged dependent variable in Eqs 2 and 3 is also a cause of endogeneity, which can be corrected using dynamic panel regression models.

To capture the endogeneity and biasedness inherent in the models, the GMM estimator by Arellano and Bover [58] and Blundell and Bond [59] was employed. Here, values of the lag-dependent variables are implored as instruments to cater for the endogeneity problem. In extant literature, both differenced GMM and SYS-GMM have received a lot of attention. However, differenced GMM has been found to be less efficient in the face of small sample size with persistent time series [60, 61], as in the case of this study. SYS-GMM outperforms difference GMM when the time series follows a random walk process, and the instruments in the level estimation are efficient predictors of the endogenous variables [59, 61]. SYS-GMM combines the standard set of moment conditions in first-difference with their lagged levels as instruments, and an additional set of moment conditions derived from the equation in levels [58, 59, 62]. A two-step SYS-GMM estimator is asymptotically more efficient compared to a one-step estimator; based on a sub-optimal weighting matrix [61]. Further, SYS-GMM is employed because [63] argued that it uses the orthoganality condition of the lag dependence variable, and first difference to the error term which suffers from potential small sample bias in fixed time periods; in a situation where the dependent variable shows high degree of persistence. SYS-GMM algorithm uses additional moment conditions, which is generated by combining the first difference of the lagged dependent variable and the sum of the cross-sectional fixed effect and the contemporaneous error term [63]. The moment conditions are strictly exogenous, in other to deal with the endogeneity problems inherent in most finance and economics data [63].

Next, the study sort to test the overall validity of the instruments implemented in GMM using Sargan test. Further, a test of whether there is no serial correlation between the error term and lagged instruments used are enough to explain the model estimated was conducted. This was done using the Arellano-Bond’s first and second-order tests of autocorrelation. In other to ensure the absence of multicollinearity in the model, the study carried out a cross-correlation analysis (refer to Table 3). Apart from aid and trade which showed a strong significant positive correlation, the rest of the variables did not show a very high correlation among themselves.

**Table 3:** Correlation matrix table.

		1	2	3	4	5	6	7	8	9	10	11	12	13	14	15
1	Women-hung															
2	Temp	0.18														
3	CF	-0.32	-0.02													
4	Adapt	-0.25	-0.01	0.911												
5	Mitigation	-0.37	-0.04	0.87	0.58											
6	Aid	-0.15	-0.05	-0.15	-0.13	-0.11										
7	FDI	-0.01	0.11	-0.01	0.15	0.29	-0.12									
8	Gov’t-Spending	-0.09	-0.01	0.15	0.23	-0.01	-0.01	0.01								
9	Population	-0.09	0.30	-0.05	0.35	0.31	-0.29	0.54	0.19							
10	GDP	-0.22	-0.01	-0.11	-0.14	0.22	0.04	0.23	-0.37	0.16						
11	COC	-0.35	-0.06	0.03	-0.01	0.04	0.46	-0.06	-0.03	-0.19	0.32					
12	Food prod	0.01	0.11	-0.13	-0.03	-0.05	0.17	0.02	0.02	-0.10	-0.09	0.07				
13	Trade	-0.15	-0.03	-0.07	-0.04	-0.09	0.84	-0.15	0.06	-0.19	0.04	0.43	0.07			
14	Agric	-0.07	0.06	0.12	0.01	0.19	-0.35	0.34	-0.07	0.34	0.14	-0.06	-0.09	-0.37		
15	Irrigation	-0.21	-0.07	0.01	-0.03	0.05	-0.02	0.03	0.01	-0.01	0.13	0.14	-0.14	0.03	0.20	

* *p* < 0.05, ** *p* < 0.01, *** *p* < 0.001

## 4 Findings

The results of both static and dynamic estimations are presented in Table 4. The main methodology employed for our analysis was SYS-GMM, and the results were triangulated with POLS and FE estimators. The robustness of our results was checked with panel quantile regression models. To ensure the validity of the instruments used in the absence of serial auto-correlation of residuals in SYS-GMM, the study performed the Sargan test of overidentifying restrictions with the Arellano–Bond test for serial correlation. The finding from Table 4 indicates that all null hypotheses were rejected. This implies that the instruments are appropriate and shows the absence of serial correlation for the residuals in second differences.

**Table 4:** Regression result for Total CF.

	SYS-GMM		FE		POLS	
VARIABLES	Model 1	Model 2	Model 1	Model 2	Model 1	Model 2
**Women-Hunger(t-1)**	0.987***	0.988***	0.897***	0.931***	0.961***	0.978***
	(0.00181)	(0.00221)	(0.0263)	(0.0111)	(0.0295)	(0.0137)
**Temperature**	0.0891***	0.0948***	0.00105	0.000741	0.0881***	0.0835**
	(0.0109)	(0.0137)	(0.00417)	(0.00426)	(0.0335)	(0.0328)
**Climate Finance**	-1.583***	-2.067***	-0.213***	-0.220***	-1.968***	-2.033***
	(0.115)	(0.164)	(0.0610)	(0.0644)	(0.329)	(0.356)
**Aid**		-0.0320***		-0.00168		-0.0246**
		(0.00519)		(0.00202)		(0.0114)
**FDI**		4.91e-10***		0		8.23e-10**
		(1.72e-10)		(5.64e-11)		(3.45e-10)
**Gov’t-Spending**		-0.173**		-0.0120		-0.222*
		(0.0792)		(0.0302)		(0.132)
**Population**	4.812***	2.849***	1.529***	2.116***	5.992***	4.465***
	(0.374)	(0.514)	(0.516)	(0.626)	(0.881)	(0.956)
**GDP per capita**	-0.000128**	-0.000554***	0.000207	0.000134	0.000193	-0.000359*
	(5.77e-05)	(9.97e-05)	(0.000173)	(0.000183)	(0.00015)	(0.000196)
**Control-of-corruption**	-34.36***	-25.44***	-0.0481	-0.644	-23.32***	-17.53***
	(1.848)	(2.481)	(2.544)	(2.596)	(4.479)	(4.596)
**Food Production**	-0.0113	-0.0162*	0.00819**	0.00729*	0.0156	0.00580
	(0.00718)	(0.00943)	(0.00388)	(0.00411)	(0.0261)	(0.0261)
**Food Import**	0.0324***	0.0627***	-0.00730	-0.00567	0.0228*	0.0463***
	(0.00407)	(0.00765)	(0.00693)	(0.00743)	(0.0121)	(0.0160)
**Agricultural Land Use**	-0.0317*	-0.0186	-0.297***	-0.308***	-0.0364	-0.0500
	(0.0170)	(0.0222)	(0.108)	(0.109)	(0.0316)	(0.0333)
**Irrigation**	-0.369***	-0.178***	-1.279***	-1.379***	-0.237***	-0.173**
	(0.0451)	(0.0580)	(0.461)	(0.473)	(0.0834)	(0.0824)
**Constant**	55.53***	65.93***	58.86***	58.99***	52.56***	59.67***
	(1.682)	(2.162)	(6.148)	(6.409)	(4.682)	(4.824)
**AR(1)**	0.6378	0.9438				
**AR(2)**	0.9600	0.4688				
**Sargan**	0.2456	0.2444				
**R** ^**2**^			0.194	0.217	0.432	0.448

Standard errors in parentheses,

*** p<0.01,

** p<0.05,

* p<0.1

**Fig 1:**
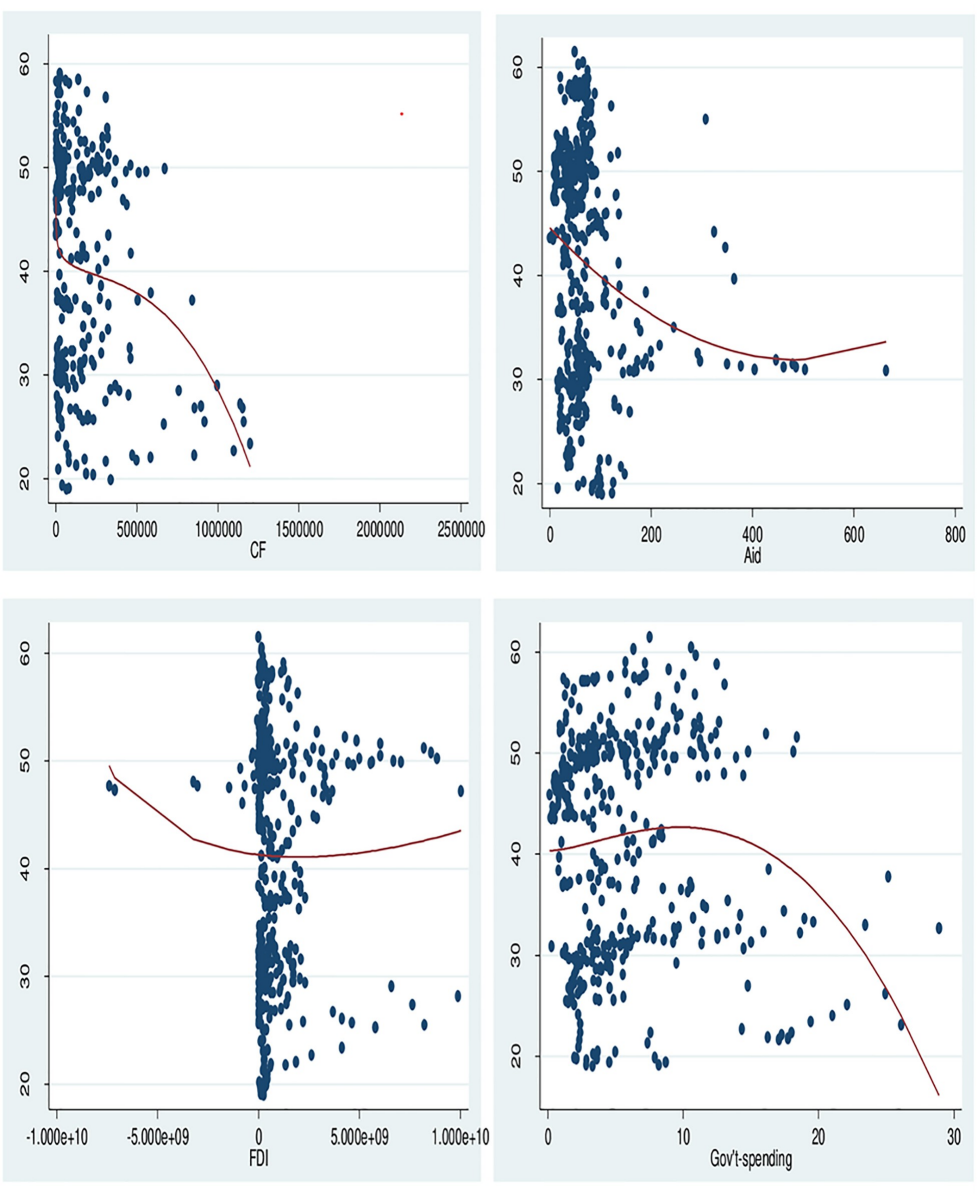
Climate finance variables and women hunger. Source: Authors Computation.

The main objective stated at the outset of this study was to assess the influence of climate finance on women-hunger in SSA. The findings of the study as presented in Table 4 points out that, climate finance significantly reduces women-hunger at 1 percent level of significance for all three models. Indicating that, a percentage increase in climate finance significantly reduces the number of hungry women in SSA by at least 1.5 percent. This shows that climate finance has an elastic impact on women-hunger reduction in SSA (Fig 1 refers); indicating that world policies targeting women-hunger and poverty will yield better results when churned through climate funds. It is no surprise because CF targeting gender has increased by 55% since 2014, hence a positive outcome. This outcome however contradicts the structural oppression theory of feminism by Freidrich Engels and Karl Marx [33]. The researchers argue that if women receive funds, their male counterparts will oppress them and take the money yielding no positive influence on them.

Next, we look at the other constituents of climate funds from the broader definition (i.e. Aid, FDI and Gov’t-spending). Aid showed a significant women-hunger alleviation effect at 1 percent level of significance (source: SYS-GMM estimates from Table 4). A unit increase in Aid reduces hunger by at least 0.01 as depicted in Fig 1. This finding is opposed to the dictates of the structural oppression theory and the findings of [32]. This points out that climate funds extended by way of financial aid and targeted at women, yields hunger alleviation among women. Thirdly, Gov’t-spending also showed a woman-hunger reduction effect at 5 percent level of significance. To show that government’s allocated contributions geared at mitigating and adapting to climate change can play a significant role in women-hunger alleviation. Sadly, FDI indicated a women-hunger worsening effect at 1 percent level of significance. The results indicate that a USD 1 million increase in FDI worsens hunger by 1.4 percent (refer to Fig 1 and Table 4). The FDI results show that private sector investment in CF may have little influence on women-hunger alleviation. This finding affirms the pollution haven hypothesis. A weak women-hunger alleviation effect by FDI is expected, because of the growing problem of agricultural feminisation among developing countries. Even though FDI breeds more formal employment, women are still forced into the informal sector such as agriculture due to childbirth and urbanisation. This choice of employment also helps them make time in taking care of the home. For effective and efficient usage of CF in developing countries where corruption abounds, there is the need to control for corruption in the analysis. Interestingly, COC indicated a significant women-hunger alleviation effect at 1 percent. Proving that, as SSA exerts more effort in fighting corruption in their CF usage, this will translate into stronger women-hunger reduction.

The study also looked at how climate change is influencing women-hunger in SSA. Temperature showed a significant women-hunger worsening effect for all models and estimators. This shows that hotter regions in SSA are more likely to suffer higher rates of women-hunger, as compared to those in colder temperates. This explains why ECOWAS region has the highest average women-hunger rate in SSA. Population growth indicated a significant women-hunger worsening effect at 1 percent level of significance. This finding affirms the Malthusian theory. The theory argues that the population growth rate will outnumber the food production rate causing a rise in hunger.

Other variables included in our model are agricultural or food production-related variables. These include food production, food import, agricultural land use and irrigation. Among the agricultural or food production variables, agricultural land use and irrigation showed significant women-hunger alleviation. By contrast, food import showed women-hunger worsening effect in SSA. Indicating that, climate funds invested in reclaiming agricultural land and improving irrigation will see improvement in women-hunger alleviation. However, climate funds or development aid extended via food import to developing countries will worsen women-hunger.

Climate finance is further segregated into mitigation and adaptation finance, and analysed accordingly. Adaptation finance is funding geared toward adjusting to the current and future unexpected harmful impact of climate change. On the other hand, mitigation finance are funds extended to countries to reduce greenhouse gas emission, to prevent or minimise the exacerbating effect of climate change. The OECD [54] report asserted that the mainstreaming of gender issues is more rampant in adaptation comparative to mitigation activities. Furthermore, the mainstreaming of gender has been largely uneven in climate-sensitive sectors. This section seeks to find out which financing mode (i.e. adaptation or mitigation financing) significantly impacts women-hunger alleviation in SSA. The findings indicate that both mitigation and adaptation finance significantly improve women-hunger alleviation (refer to Table 5). Yet, adaptation finance is more efficient than mitigation finance in alleviating women-hunger. This affirms the OECD [54] report, and also shows that much climate finance is extended to areas already experiencing the exacerbating effect of climate change.

**Table 5:** Regression result for mitigation and adaptation finance.

	MITIGATION			ADAPTATION		
VARIABLES	SYS-GMM	FE	POLS	SYS-GMM	FE	POLS
**Women-Hunger(t-1)**	0.989***	0.897***	0.921***	0.986***	0.865***	0.911***
	(0.00231)	(0.0121)	(0.0265)	(0.00222)	(0.0211)	(0.0285)
**Temperature**	0.00399***	0.00425	0.119***	0.00286***	0.00131	0.0866***
	(0.00119)	(0.00640)	(0.0336)	(0.000973)	(0.00422)	(0.0330)
**MITIGATION/ADAPT**.	-0.8279***	-0.185***	-0.984***	-1.0672***	-0.205***	-1.552***
	(0.0705)	(0.0458)	(0.192)	(0.1004)	(0.0542)	(0.298)
**Aid**	-0.00100***	-0.00352	-0.0149	-0.00158***	-0.00164	-0.0252**
	(0.000318)	(0.00234)	(0.00904)	(0.000323)	(0.00200)	(0.0114)
**FDI**	-9.3e-11***	-0	6.75e-10**	-9.32e-11***	0	6.61e-10*
	(0)	(7.30e-11)	(2.92e-10)	(0)	(5.56e-11)	(3.41e-10)
**Gov’t-Spending**	-0.0126**	-0.0312	-0.307***	0.00881*	-0.0245	-0.257*
	(0.00509)	(0.0315)	(0.101)	(0.00496)	(0.0302)	(0.132)
**Population**	-0.132***	2.135***	4.056***	-0.00180	2.058***	4.215***
	(0.0328)	(0.446)	(0.752)	(0.0324)	(0.620)	(0.955)
**GPCY**	3.37e-05***	-0.000507***	-0.00043**	5.86e-05***	0.000310	-0.00053***
	(6.68e-06)	(0.000182)	(0.000173)	(5.89e-06)	(0.00018)	(0.000195)
**Control-of-corruption**	-0.335*	6.589**	-15.92***	-0.417**	-1.030	-17.75***
	(0.186)	(2.677)	(3.958)	(0.169)	(2.568)	(4.548)
**Food Production**	0.00465***	0.0126***	-0.0211	0.00644***	0.00757*	0.00505
	(0.000709)	(0.00469)	(0.0225)	(0.000616)	(0.00404)	(0.0262)
**Food Import**	0.00190***	-0.00717***	0.0170**	0.00354***	-0.00642	0.0448***
	(0.000358)	(0.00271)	(0.00813)	(0.000485)	(0.00734)	(0.0161)
**Agricultural Land Use**	0.00399***	-0.465***	-0.0671**	0.00409***	-0.348***	-0.0539
	(0.00152)	(0.0667)	(0.0286)	(0.00145)	(0.106)	(0.0335)
**Irrigation**	-0.00137	-1.254***	-0.165**	-0.00416	-1.589***	-0.190**
	(0.00421)	(0.341)	(0.0683)	(0.00377)	(0.468)	(0.0820)
**Constant**	-0.752***	66.68***	49.71***	-0.923***	61.09***	55.01***
	(0.188)	(4.094)	(3.515)	(0.180)	(6.336)	(4.529)
**AR(1)**	0.1959			0.0894		
**AR(2)**	0.618			0.2417		
**Sargan**	0.9963			0.9859		
**R** ^**2**^		0.358	0.414		0.225	0.440

Standard errors in parentheses,

*** p<0.01,

** p<0.05,

* p<0.1

### 4.1 Sensitivity analysis

As proposed by [64] and [65], the study further tested the robustness of the static and dynamic panel regression results using fixed effect panel quantile regression estimates to control for distributional heterogeneity. The traditional regression estimator focuses on the mean effect, which may cause over or under-estimation of the relevant coefficients or even lose the ability to detect important relationships [66, 67]. Quantile regression uses a generalization of median regression analysis to other quantiles. Following the study by [67], we specify the fixed effect panel quantile regression model as:

$$Q_{y_{it}}\left(\tau_{k}ǀ{\alpha_{i},x}_{it}\right)=\alpha_{i}+x_{it}^{l}\beta (\tau_{k})$$

A major setback affecting the panel fixed effect quantile regression estimator is the inclusion of a considerable number of fixed effects (*α*_*i*_), subject to the incidental parameters problem [67–69]. To circumvent this problem, [65] and [67] proposed the treatment of unobservable fixed effects as parameters to be jointly estimated with the covariate effects for different quantiles. What this method seeks to do is to introduce a penalty term in the minimization to address the computational problem of estimating a mass of parameters. In Table 6, we present the 25^th^, 50^th^, 75^th^ and 90^th^ quantiles of the conditional women-hunger distribution.

**Table 6:** Fixed effect panel quantile regression.

VARIABLES	25^th^	50^th^	75^th^	90^th^
**Temperature**	0.0471	0.841	1.286*	1.416***
	(1.149)	(0.736)	(0.728)	(0.498)
**Log of Climate finance**	-1.615	-0.801	-0.109*	-0.369
	(1.067)	(0.590)	(0.866)	(0.570)
**Log of Mitigation**	-0.511	-0.206	-0.118	-0.0801*
	(0.632)	(0.438)	(0.241)	(0.237)
**Log of Adaptation**	-0.724	-0.683	-0.534	-0.305
	(0.781)	(0.492)	(0.589)	(0.393)
**Aid**	-0.0165	-0.0292***	-0.0111	-0.00724
	(0.0174)	(0.00989)	(0.0146)	(0.00963)
**FDI**	1.36e-09*	4.14e-10	1.27e-10	7.37e-11
	(7.32e-10)	(4.54e-10)	(2.84e-10)	(2.06e-10)
**Gov’t-Spending**	-0.243	-0.374**	-0.179	-0.0681
	(0.281)	(0.168)	(0.166)	(0.123)
**Population**	5.686***	2.294	2.626***	3.023***
	(2.129)	(1.561)	(0.943)	(0.657)
**GDP per capita**	-0.000141	-0.000526**	-0.000628***	-0.000563***
	(0.000374)	(0.000222)	(9.98e-05)	(8.61e-05)
**Control-of-corruption**	-19.89***	-19.94***	-15.70*	-12.33**
	(5.711)	(5.423)	(8.223)	(6.076)
**Food Production**	0.0185	-0.00473	-0.00405	-0.0237
	(0.0324)	(0.0170)	(0.0213)	(0.0231)
**Food Import**	0.0507**	0.0588***	0.0192	0.00344
	(0.0242)	(0.0145)	(0.0189)	(0.00905)
**Agricultural Land Use**	-0.0506*	0.0479	0.0301*	-0.00378
	(0.0888)	(0.0453)	(0.0563)	(0.0406)
**Irrigation**	-0.0779	-0.227**	-0.264***	-0.284***
	(0.287)	(0.100)	(0.0647)	(0.0573)
**Constant**	57.63*	40.17*	22.23	21.30*
	(33.28)	(21.26)	(17.60)	(12.24)

Standard errors in parentheses,

*** p<0.01,

** p<0.05,

* p<0.1

Fixed effect panel quantile regression estimates presented in Table 6 are consistent with the GMM, fixed effect and POLS estimates. For instance, almost all the climate finance variables-climate finance, mitigating and adaptation finance (i.e. aid, FDI, COC and Gov’t-spending)-showed homogenous effect on women-hunger alleviation from the 25^th^ to the 90^th^ percentile, although non-significance for most variables. In addition, the climate change variable (i.e. temperature) also exhibit homogenous effect on women-hunger alleviation for all quantiles. For the agricultural or food production variables, they all showed homogenous effect on women-hunger for all percentiles apart from AGL which showed a heterogenous effect on women-hunger. In all, the panel fixed effect quantile regression result strongly reinforces the main regression results of the study.

## 5. Conclusion

This study was set on the road of finding out how CF influences women-hunger alleviation, using an unbalanced panel of 43 SSA countries for the period 2006–2018. Data collated was analysed using SYS-GMM and complimented with panel fixed effect and POLS estimations. A major setback to these traditional estimation techniques is that they focus on the mean effect, which is likely to cause over or under-estimation of the relevant coefficients. In response to this, panel fixed effect quantile regression was carried out as sensitivity analysis to ensure that median regression analysis is extended to other quantiles. The findings showed that CF (narrow definition of CF) and broad definition variables of CF (aid and Gov’t-spending) have a positive potential of alleviating women-hunger in SSA. This is contrary to the expectations of the structural oppression theory of feminism. However, private financing of CF via FDI have a weaker potential of alleviating women-hunger in SSA. In addition, both mitigation and adaptation financing have a very good potential in alleviating women-hunger. Further, SSA countries strengthening their quest to fight against corruption are more likely to experience women-hunger alleviation. For the climate variables, warmer areas in SSA have a higher potential to experience worsened conditions of women-hunger.

It can be seen from the findings that the gender requirement included in CF extended to developing countries is seeing a positive women-hunger alleviation. Based on the findings, the study recommends that CF should be extended as financial aid or supporting government budget, due to its potential in alleviating women-hunger. Secondly, SSA countries should strengthen their quest in controlling corruption in other to ensure the appropriate use of CF to achieve women-hunger alleviation. Thirdly, much CF should be extended to warmer areas of SSA due to the exacerbating effect of temperature on women-hunger. A major limitation of our study was how the women-hunger variable is estimated. Thus, future research can look at other dimensions of both women-hunger and climate finance to triangulate the literature.
